# Vitamin‐C‐dependent downregulation of the citrate metabolism pathway potentiates pancreatic ductal adenocarcinoma growth arrest

**DOI:** 10.1002/1878-0261.13616

**Published:** 2024-02-29

**Authors:** Aiora Cenigaonandia‐Campillo, Ana Garcia‐Bautista, Anxo Rio‐Vilariño, Arancha Cebrian, Laura del Puerto, José Antonio Pellicer, José Antonio Gabaldón, Horacio Pérez‐Sánchez, Miguel Carmena‐Bargueño, Carolina Meroño, Javier Traba, María Jesús Fernandez‐Aceñero, Natalia Baños‐Herraiz, Lorena Mozas‐Vivar, Estrella Núñez‐Delicado, Jesús Garcia‐Foncillas, Óscar Aguilera

**Affiliations:** ^1^ Translational Oncology Division, Oncohealth Institute IIS‐Fundación Jimenez Diaz‐UAM (Madrid) Spain; ^2^ Molecular Recognition and Encapsulation Research Group (REM), Health Sciences Department Universidad Católica de Murcia (UCAM) Spain; ^3^ Bioinformatics and High‐Performance Computing Research Group (BIO‐HPC), Computer Engineering Department Universidad Católica de Murcia (UCAM) Spain; ^4^ Centro de Biología Molecular Severo Ochoa, Consejo Superior de Investigaciones Científicas Universidad Autónoma de Madrid (CSIC‐UAM) Spain; ^5^ Instituto Universitario de Biología Molecular‐UAM (IUBM‐UAM), Departamento de Biología Molecular Universidad Autónoma de Madrid Spain; ^6^ Hospital Clínico San Carlos (HCSC) Madrid Spain; ^7^ Preclinical programe START Madrid‐FJD Hospital fundación Jiménez Díaz Spain; ^8^ Universidad Católica de Murcia (UCAM) Spain

**Keywords:** citrate synthase, gemcitabine, metabolism, PDAC, vitamin C

## Abstract

In pancreatic ductal adenocarcinoma (PDAC), metabolic rewiring and resistance to standard therapy are closely associated. PDAC cells show enormous requirements for glucose‐derived citrate, the first rate‐limiting metabolite in the synthesis of new lipids. Both the expression and activity of citrate synthase (CS) are extraordinarily upregulated in PDAC. However, no previous relationship between gemcitabine response and citrate metabolism has been documented in pancreatic cancer. Here, we report for the first time that pharmacological doses of vitamin C are capable of exerting an inhibitory action on the activity of CS, reducing glucose‐derived citrate levels. Moreover, ascorbate targets citrate metabolism towards the *de novo* lipogenesis pathway, impairing fatty acid synthase (FASN) and ATP citrate lyase (ACLY) expression. Lowered citrate availability was found to be directly associated with diminished proliferation and, remarkably, enhanced gemcitabine response. Moreover, the deregulated citrate‐derived lipogenic pathway correlated with a remarkable decrease in extracellular pH through inhibition of lactate dehydrogenase (LDH) and overall reduced glycolytic metabolism. Modulation of citric acid metabolism in highly chemoresistant pancreatic adenocarcinoma, through molecules such as vitamin C, could be considered as a future clinical option to improve patient response to standard chemotherapy regimens.

## Graphical abstract

Citrate metabolism, crucial for pancreatic cancer proliferation, is targeted by vitamin C, potentiating gemcitabine‐induced growth arrest. Ascorbate inhibits citrate synthase, diminishing glucose‐derived citrate levels. Ascorbic acid exposure not only reduces citrate generation but also targets catabolism, downregulating *ACLY* and *FASN* expression. This reduction in citric acid metabolism correlates with decreased glycolytic metabolism. Vitamin C‐induced metabolic change synergizes with gemcitabine.

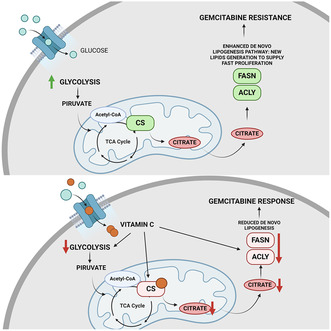

**Abbreviations**
ACLY: ATP citrate lyase
CS: citrate synthase
FASN: fatty acid synthase
GEM: gemcitabine
GLUT1: glucose transporter 1
GTEx: The Genotype Tissue Expression project
IC50: inhibitory concentration 50
IHC: immunohistochemistry
LDHA: lactate dehydrogenase
PDAC: pancreatic ductal adenocarcinoma
PDH: pyruvate dehydrogenase
PDK1: pyruvate dehydrogenase kinase 1
PDX: patient‐derived xenograft
shRNA: short hairpin RNA
SREBP1: sterol regulatory element binding protein 1
TGCA: The Cancer Genome Atlas
VC: vitamin C

## 1 Introduction

Resistance in Pancreatic Ductal Adenocarcinoma (PDAC) arises as one of the most serious challenges for the scientific community nowadays. Despite great effort has been done, none of the standard current therapies is curative for pancreatic cancer and resistance rapidly appears [1].

Pancreatic Ductal adenocarcinoma (PDAC) is the most aggressive type of pancreatic cancer worldwide with an unfortunate 5‐year survival rate of 8% [2]. Current therapeutic options for PDAC rely on standard chemotherapy regimens. Gemcitabine (Gemzar^®^) has been the standard of care for first‐line treatment in pancreatic cancer for decades, improving patients' overall survival for 5 months [3]^.^

Cancer metabolism plays a central role in therapy resistance in PDAC. Metabolic rewiring has been considered a hallmark in cancer and has been closely associated with gemcitabine resistance in pancreatic tumors. Some Warburg metabolic partners together with augmented *de novo* fatty acid synthesis have been directly associated with gemcitabine resistance in pancreatic cancer [4, 5].

Synthesis of the biological precursors necessary for the process of cancer cell growth, tumor proliferation, and membrane biogenesis needs enhanced production of cholesterol and fatty acids, then requiring large amounts of citrate in tumor cells as substrate.

In absence of extracellular citrate, cancer cells employ different mechanisms to support mitochondrial Citrate Synthase (CS) activity and, in turn, increased citrate synthesis. Contrary to normal cells, mostly relying on dietary fat, more than 90% of triacylglycerol fatty acids present in cancer cells are *de novo* synthesized from mitochondrial citrate [6], which is the intermediate metabolite between cytosolic and mitochondrial acetyl coenzyme A (CoA) [7].

Mitochondrial Citrate Synthase plays a bridging role connecting the Warburg glycolytic metabolism with the synthesis of new lipids. Inside the mitochondria, glucose‐derived pyruvate is converted into acetyl‐CoA by pyruvate dehydrogenase, which is then transformed into citrate via CS. Mitochondrial citrate is exported to the cytoplasm, enabling extra mitochondrial production of acetyl‐CoA for generation of new fatty acids [6]. Obviously, increasing of both availability of intracellular citrate and CS enzyme activity are directly associated and have been reported in pancreatic cancer [8].

Moreover, apart from CS, many other enzymes participating in citrate metabolism towards *de novo* fatty acids synthesis are upregulated in pancreatic ductal adenocarcinoma, including ATP citrate lyase (ACLY), fatty acid synthase (FASN), stearoyl‐CoA desaturase (SCD1) and 3‐hydroxy‐3‐methylglutaryl coenzyme A reductase (HMGCR) [9].

Close relationship between activity and expression of some enzymes involved in *de novo* lipid synthesis and chemotherapy resistance has been also reported. For example, in 2017 Saer Tadros *et al*. [10] reported that elevated *de novo* lipogenesis correlates with reduced response to gemcitabine and inhibition of the pathway results in synergistic effects on gemcitabine treatment.

Interestingly, despite the importance of citrate metabolism in the production of lipids and the high activity of mitochondrial Citrate Synthase observed in this type of tumors, no scientific evidence has been published regarding the importance of this citrate availability in the chemoresistance of pancreatic adenocarcinoma to standard gemcitabine regimens and tumor proliferation.

Vitamin C or ascorbic acid is an essential micronutrient for humans and plays a key role in several crucial biochemical reactions. Reduced levels of Ascorbic Acid have been described in many chronic diseases such as cancer, immune diseases, arteriosclerosis or depression [11].

Dr. Ewan Cameron and Dr. Linus Pauling performed the first clinical trial in early 1970s treating advanced cancer patients with intravenous high doses of vitamin C. They reported more than 50 cases showing some interesting clinical outcomes [12]. Since then, ascorbate administration as an anticancer agent has been controversial during years. Double blind placebo‐controlled trials showed no effect. However, these studies used oral vitamin C administration, where antineoplasic plasma concentrations cannot be reached in patients. Some investigations concluded intravenous administration to be necessary for vitamin C to have antitumor activity [13]. Since then, several phase I and phase II clinical trials have been performed using pharmacological doses of vitamin C, in combination with several chemotherapeutic agents, testing the safety and efficacy of vitamin C in different neoplasias intravenously [14]. However, the exact role and molecular mechanisms underlying the activity of vitamin C in synergy with chemotherapeutic agents in oncological disease is not yet well understood and is subject of hot scientific debate.

In 2015 Yun *et al*. [15] published a groundbreaking scientific article concluding that vitamin C selectively kills KRAS mutant colorectal cancer (CRC) cell lines targeting aberrant glucose metabolism. In 2021, these statements were supported by previous data of our laboratory displaying that, in fact, ascorbic acid induces HIF1α degradation, leading to reduced GLUT1 and PDK1 expression, finally increasing PDH activity in KRAS mutant CRC [16]. Since then, it has been reported that vitamin C is able to target WNT signaling and glycolysis in pancreatic cancer [17]. Moreover, phase II clinical trials have described ascorbate's ability to target EMT in PDAC together with gemcitabine reducing metastatic capacity [18].

In this work we describe an unexpected metabolic role of vitamin C in citrate metabolism pathways in the context of pancreatic cancer. Pharmacological treatment of vitamin C led to inhibition of CS activity, reducing glucose conversion into citrate. Moreover, FASN and ACLY were also downregulated through AKT/SREBP1 axis which deregulated citrate metabolism through *de novo* lipogenesis. Reduced availability of citrate to be metabolized upraised gemcitabine efficacy in Mia‐PaCa2 and CRL‐2558 cells, which has been validated in a PDAC PDX model.

Dismissed citrate metabolism correlated with reduced Warburg effectors such as the pyruvate dehydrogenase kinase 1 (PDK1) and the glucose transporter 1 (GLUT1), while pyruvate dehydrogenase activity was enhanced upon ascorbate treatment. Reduced LDHA expression was also observed after vitamin C exposure, leading to a vast extracellular acidification rate (ECAR) reduction.

## 2 Materials and methods

### 2.1 Cell culture

Two human‐derived PDAC cell lines obtained from the American Type Culture Collection (ATCC), CRL‐2558 (RRID: CVCL_3567) and Mia‐PaCa2 (RRID: CVCL_0428), as well as HEK 293T (RRID: CVCL_0063) cells, were cultured in DMEM medium supplemented with 10% foetal bovine serum (FBS) (both from Invitrogen, Waltham, MA, USA), HEPES (25 mm) and Penicillin (100 U·mL^−1^), Streptomycin (100 U·mL^−1^) at 37 °C with 5% CO_2_. All cells were tested for mycoplasma every 30 days. Identity of all cell lines has been authenticated using STR profiling.

### 2.2 Cell treatment

3 × 10^6^ CRL‐2558 and Mia‐PaCa2 cells were counted using a Trypan Blue reduction assay (Gibco, Grand Island, NY, USA) and seeded in flask and left to attach overnight before treatment.

#### 2.2.1 Nickel chloride treatment

NiCl_2_ was added to the medium at 500 μm to stabilize HIF1α and induce hypoxia. Cells were collected after 24 h.

#### 2.2.2 Gemcitabine treatment

Gemcitabine was added at 2.5 μm to the cell medium and the cells were collected after 24 h. NiCl_2_ was added prior 2 h Gemcitabine.

#### 2.2.3 Vitamin C treatment

The sodium ascorbate stock (95% purity) was diluted in 50 mL of milliQ water to obtain a concentration of 100 mm. 3 mm of ascorbate was administrated to the medium and collected them after 6 h prior gemcitabine and NiCl_2_ treatment completion.

### 2.3 *In vivo* studies

KRAS mutation harboring pancreatic cancer Patient‐derived Xenograft model was established. In brief, 2 mm^3^ tumors fragments were subcutaneously implanted in 5–6 week old female athymic nude NU(NCr)‐Foxn1^nu^ mice (Charles River). Tumor size was measured twice a week with digital calipers and tumor volume was calculated using the formula (Length × Width^2^ × /2) The animals were randomized into different treatment groups when the tumors reached 150–200 mm^3^. Animals were treated with vehicle, GEM (30 mg·kg^−1^, twice a week, i.p.), vitamin C (4 g·kg^−1^, daily, i.p.) and a combination of GEM and vitamin C.

Animals were housed in the Animal Model Core Facility of IIS–Fundacion Jimenez Diaz (ES28079000089). Mice were accommodated in individually ventilated cages (5 mice per cage), including a wire rack in the cage for holding food and a water bottle. Animals were housed on a 12‐h light/dark cycle. All animal procedures and experimental protocols were approved by the Ethical Animal Research Committee at IIS‐Fundacion Jimenez Diaz (Madrid, Spain) and were also conducted in accordance with institutional standards (Reference n°: PROEXP 142–17) (IRB nª PIC124‐21_FJD_JESUS GARCIA FONCILLAS), which fulfilled the requirements established by the Spanish government and the European Community (Real Decreto R.D. 53/2003).

### 2.4 *CS* and *FASN* knockdown cell generation

To generate *CS* and *FASN* knockdown cells the vector pLV[shRNA]‐Puro‐U6>hCS[shRNA#1] was purchased from VectoBuilder and pLKO.1 puro‐humanU6‐shRNA FASN was a gift from Elizabeth Stoll (Addgene plasmid # 82327; http://n2t.net/addgene:82327; RRID:Addgene_82327). The empty pLKO and Plv vectors were a gift from Dr. Pedro Mateos (Universidad de Alcala de Henares). Bacteria harboring insert or empty vectors were grown in LB media supplemented with 50 μg·mL^−1^ Ampicillin for 16 h at 37 °C and shaking. Plasmid DNA was extracted and purified using maxiprep protocol (QIAGEN Plasmid Maxi Kit, ref. 12162, Venlo, Netherlands). HEK293T cells were seeded and cultured to 80% confluence. Cells were transfected with 10 μg of expression vector and 10.5 μg of viral packaging plasmids (3 μg of pVSVG, 5 μg of RRE and 2.5 μg of RRV) using 1 mg·mL^−1^ polyethyleneimine (PEI) in 3 : 1 ratio with respect to DNA quantity. Media was replaced after 16 h post transfection. 48 h later, the media was collected and filtered with 0.44 nm filters (Fisher brand™ ref. 15216869, Waltham, MA, USA) and then supplemented with 10 μg·μL^−1^ polybrene diluted 1 : 1000. For infection, the conditioned media containing the viral particles was added to the indicated cell lines and renewed periodically every 2 h for 8 h total. After 24 h, puromycin (P8833‐10 MG) specific for Puro‐ Barcode‐based vectors was added to select the infected cell lines over a total of 72 h.

### 2.5 Proliferation assay

Proliferation assay was performed using crystal violet staining. In brief 3 × 10^4^ well were seeded in 24‐well plate and incubated for 72 h. Cells were fixed using cold methanol for 10 min and stained with crystal violet for further 15 min. Obtained color was solubilized adding 1% SDS and read at 570 nm.

### 2.6 Citrate levels

Citrate levels were measured in frozen PDX generated tumor tissue using Abcam ab83396 kit (Cambridge, UK) according to manufacturer's instructions. 20 mg of tissue was harvested and homogenized using a dounce homogenized. Samples were deproteinized using Abcam ab204708 kit according to manufacturer's instructions.

### 2.7 Inhibition growth (IG) assays

IG assays were performed with the Cell Counting Kit‐8 (CCK‐8; Sigma‐Aldrich, San Luis, MO, USA). Between 3 and 10 × 104 cells per well were seeded in a 24‐well plate and incubated for 24 h. Then, gemcitabine was added to the plate in a concentration range from 0.1 to 50 μm. 72 h later, CCK‐8 was added to each well and incubated for 2 h at 37 °C and 5% CO_2_. The absorbance was measured at 450 nm in a spectrometer plate reader. Each experiment was developed in triplicate and mean value for absorbance data in each concentration point was used for calculations. The IG50 value for each cell line was obtained by a function which fits a 5‐parameter logistic model: *y* = *B* + (*T* − *B*)/[1 + 10b(*x*mid‐*x*)]*S*. where *B* and T are the lower and upper asymptotes, *b* is the slope, *x*mid is the value of *x* at which the inflection point occurs, and s is a coefficient of skewness. Curves were developed by plotting the mean values derived from the experimental triplicates as described in earlier laboratory articles [19].

### 2.8 Western blot

CRL‐2558 and Mia‐PaCa2 were trypsinized and washed with cold PBS then cells were lysed in cold RIPA buffer supplemented with 1× protease and phosphatase inhibitor cocktail (Roche, Basil, Switzerland). Lysates were kept on ice for 30 min vortexing every 5 min. These extracts were collected and protein contents were quantified by a BSA protein quantification assay (Thermo Fisher, Waltham, MA, USA). 20 μg of protein sample were diluted in Laemmli Buffer supplemented with β‐Mercaptoethanol. Then samples were fractioned by a SDS/PAGE gel electrophoresis and transferred to nitrocellulose membranes (1620115, Biorad, Hercules, CA, USA) or PVDF membranes (1620177, Biorad). Then, membranes were blocked incubating for 1 h in 5% milk and proteins were detected using specific primary antibodies for HIF1α (ab51608; Abcam), GLUT1 (ab115730; Abcam), PDK1 (ab110025, Abcam), P‐PDH (ab177461, Abcam), PDH (ab110334, Abcam), Citrate Synthetase (ab96600, Abcam), LDHA (ab47010, Abcam), FASN (ab128870, Abcam), ACLY (ab40793, Abcam), AKT (pan) (C67E7, Cell Signaling, Danvers, MA, USA), p‐AKT (9271, Cell Signaling), SREBP1 (nb‐600582, Novus Biological, Centennial, CO, USA) and β‐actin (A5441‐,2ML; Abcam). Horseradish peroxidase linked donkey anti‐rabbit IgG (NA934V) or sheep anti‐mouse (NA931V) antibodies (GE‐Healthcare, Chicago, IL, USA) were used as secondary antibodies. Blots were developed with the Amersham ECL Western Blotting Detection Reagent (GE Healthcare).

### 2.9 Apoptosis assays

CRL‐2558 y Mia‐PaCa2 cells were treated with NiCl_2_ (500 μm) and Gemcitabine (0.5 μm) for 24 h and vitamin C (3 mm) for 6 h, and then replaced with normal DMEM supplemented with 10% fetal bovine serum (FBS). As described in earlier articles [20], 1–5 × 10^5^ cells were collected by centrifugation and resuspended in 500 μL of 1× binding buffer. Afterwards, 5 μL of annexin V‐FITC and 5 μL of propidium iodide were added and incubated for 5 min in the dark. V‐FITC binding was analyzed by flow cytometry using a BD FACS Canto II device (Becton, Dickinson and Company, Franklin Lakes, NJ, USA) (Ex = 488 nm; Em = 350 nm) using FITC signal detector and PI staining by the phycoerythrin emission signal detector.

### 2.10 ATP and mitochondrial membrane potential assay

Mia‐PaCa2 and CRL‐2558 cells were cultivated in P96‐well plate and treated with NiCl_2_ (500 μm), Ascorbate (3 mm), gemcitabine (1 μm) or combination for 6 h. ATP determination was carried out using an ATP Luminescent assay from Abcam. Mitochondrial membrane potential was measured using Abcam 113850 kit following manufacturer's instructions.

### 2.11 Mitochondrial isolation

Six million Mia‐Paca 2 and CRL‐2558 cells were seeded in flasks and left to attach overnight. The two cell lines were treated with NiCl_2_, vitamin C and/or gemcitabine and collected for mitochondrial isolation. Mitochondria were isolated using Abcam ab110170 kit following manufacturer's instructions. VDAC1 (ab15895, Abcam) mitochondrial marker was used in blots as loading control.

### 2.12 Immunohistochemistry assays

The paraffin‐embedded sections were deparaffinized by incubating at 65 °C for 1 h. Then, antigen retrieval was performed in low pH buffer at 95 °C for 20 min. They were blocked with 3% H_2_O_2_ in TBS for 10 min. After washing, they were incubated firstly with FASN (ab128870, Abcam) dilution 1 : 100, ACLY (ab40793, Abcam) dilution 1 : 100, PDK1 (Abcam, ab110025) dilution 1 : 50, LDHA (Abcam, ab47010 1 : 100) and GLUT‐1 (Abcam, ab652) dilution 1 : 1000 for 1 h at RT, followed by incubation with the appropriate anti‐ Ig horseradish peroxidase‐conjugated polymer (EnVision, Dako, Nowy Sacz, Poland) for 20 min. The sections were developed with diaminobenzidine as a chromogen for 4 min and counterstained with hematoxylin.

### 2.13 TGCA and GTEX data analysis

FASN, AKT and SREBP1 pancreatic tumor versus healthy tissue transcript levels comparison was made using gepia 2.0 software [21]. TCGA and GTEx databases were used for analysis. The correlation between glycolytic and lipogenesis gene signature was carried out by analysis pancreatic tumor TGCA analysis.

### 2.14 *In vitro* kinetic assays

#### 2.14.1 Experimental procedure for lactate dehydrogenase

The reaction medium was composed of sodium phosphate buffer 100 mm pH 7.0, pyruvate 250 μm, NADH 10 μm, ascorbic acid at increasing concentrations (0–20 mm) and lactate dehydrogenase at 25 μg·mL^−1^. Sodium phosphate and lactate dehydrogenase were purchased from Sigma‐Aldrich (Madrid, Spain), pyruvate and NADH were from Spinreact (Barcelona, Spain).

Lactate dehydrogenase activity was followed spectrophotometrically in a Shimadzu model UV‐1603 spectrophotometer (Shimadzu‐NAGEL, Düren, Germany) at the maximum absorption of the product at 340 nm (ε_340_ = 4925 m ^−1^ cm^−1^). One unit is defined as the amount of enzyme that will oxidize 1.0 μmol NADH per min at 25 °C. The experiment was carried out at 25 °C.

### 2.15 qPCR

RNA isolation from cellular pellets was performed using the commercial kit NucleoSpin® RNA (Shimadzu‐NAGEL, Düren, Germany) according to manufacturer's instructions and described in earlier articles of the laboratory [19], and quantified with NanoDrop 2000 spectrophotometer (Thermo Fisher‐Scientific, Waltham, MA, USA). cDNA was synthesized with High Capacity cDNA Reverse Transcription kit (Applied Biosystem, Foster City, CA, USA) from 1 μL of 200 ng·μL^−1^ of total RNA following manufacturer's instructions.

Applied Biosystems 7500 Fast Real‐Time PCR System was used to perform quantitative real‐time PCR (Foster City, CA, USA). PCR reaction mix was prepared with 0.25 μL of each TaqMan probe, using the problem probe FAM marked, and housekeeping probe VIC marked, 5 μL of Universal PCR Master Mix (Applied Biosystems) and 3.5 μL of nuclease‐free water. Previously generated cDNA was added to each well, in a proportion 9 μL of mix and 1 μL of cDNA, analyzing a triplicate for each sample or condition in a 96‐well plate.

The genes explored were SLC2A1 (Hs00892681_m1), PDK1 (Hs01561847_m1), LDHA (Hs00184500_m1) FASN (Hs01005622_m1), and all of them were normalized using RPLP0 (Hs99999902_m1) (Applied Biosystems).

Each experiment was developed in triplicate and expressed as mean ± standard deviation (SD).

### 2.16 Pyruvate dehydrogenase (PDH) and citrate synthase (CS) activity assays

PDH activity was determined using the PDH Activity Colorimetric Assay Kit (Biovision, Mountain View, CA, USA) according to the manufacturer's instructions. Briefly 3 × 10^6^ cells were seeded and pre‐treated with NiCl_2_ (500 μm), Gemcitabine (0.5 μm) along 24 h and ascorbate (3 mm) along 6 h for both cell lines. Cells were lysed in PDH buffer and PDH activity was measured by the kinetic reduction of NAD^+^ to NADH+H^+^, which resulted in a colorimetric (450 nm) product proportional to the enzymatic activity present. CS activity was quantified using a CS Activity Colorimetric Assay Kit (BioVision, Milpitas, CA, USA), according to the manufacturers' instructions at the same conditions. Cells were treated with 500 μm N‐acetylcysteine (NAC) (Sigma‐Aldrich, A9165) after ascorbate addition to asses ROS‐dependent activity on citrate synthase.

### 2.17 Glycolytic rate analysis

The extracellular acidification rate (ECAR) was assessed by the Seahorse XF96 Flux Analyzer (Seahorse Bioscience, Agilent, Santa Clara, CA, USA). In brief, 2 × 10^4^ cells of Mia‐PaCa2 and CRL‐2558 cells were seeded in an incubation plate as the protocol indicated. Cells were cultures at 37 °C overnight for adhesion. Then, cells were treated with NiCl_2_ (0.5 mm) and Gemcitabine (0.5 μm) for 24 h and Vitamin C (3 mm) for 6 h. Before detection, culture medium was replaced with assay media. The glycolytic rate test kit (Seahorse Cat. #103344‐100) was purchased for ECAR detection. The assays were performed using the manufacturer protocols.

### 2.18 Metabolite analysis

For ^13^C glucose tracing, 250 000 Mia‐Paca 2 and CRL‐2558 cells were plated on 6‐well plates containing DMEM (ThermoFisher, Waltham, MA, USA) with 5.5 mm Glucose, 2 mm Glutamine, and no pyruvate. To induce hypoxia, 0.5 mm NiCl_2_ was added to the cells for 24 h. The protocol used is extensively described in previously published articles [22]. Four hours before treatment completion, the wells were washed once in PBS and then refilled with 1.5 mL DMEM, either containing 5.5 mm U–^13^C Glucose or 5.5 mm U–^12^C Glucose. Simultaneously, cells were treated with 3 mm ascorbate for 4 h.

After treatment completion, samples were collected by retrieving the media and harvesting the cells on ice. Once the media was removed, wells were washed once in ice‐cold saline solution (9 g·L^−1^ NaCl), after which the cells were scraped in 300 μL of ice‐cold extraction buffer (80% methanol, 2 μm d27 myristic acid) and transferred to a 1.5 mL tube, which was stored overnight at −80 °C.

The cellular debris was pelleted (20 000 ***g***, 15 min, 4 °C), and the supernatant was transferred to a new vial and again centrifuged (20 000 ***g***, 15 min, 4 °C). The supernatant was set aside for analysis by mass spectrometry. Protein content of the cell pellet was determined by BCA assay (ThermoFisher Scientific) for metabolite normalization.

For the mass spectrometry analysis, 10 μL of the sample was separated on an ion‐pairing liquid chromatography column, and the metabolites were resolved on a Q Exactive™ Hybrid Quadrupole‐Orbitrap™ Mass Spectrometer in negative ion mode with ESI settings (40 sheet gas flow rate, auxiliary gas flow rate 10, spray voltage of 4.8 kV, S‐lens RF level of 60, and the capillary temperature at 300 °C). A full scan (resolution set at 140 000 at 200 *m*/*z*, AGC at 3e6, 512 ms ion fill time, and 70–1050 *m*/*z* scan range) was applied.

Metabolites were quantified according to their elution time and *m*/*z* ratios using the open‐source analysis platform EL‐MAVEN. Coupled processing of the data in POLLY™ integrated correction of naturally occurring carbon isotopes. Metabolite abundances were normalized to protein content.

Pyruvate was analyzed using MALDI and direct infusion of the sample in scimaX (Bruker, Billerica, MA, USA) instrument based on the following parameters: laser power 55 Ip, 500 laser shots, frequency 2000 Hz with ultra large focus and enabled smartwalking with random pattern. We accumulated up to 100 scans. Metabolite analysis was done using mmass open‐source software.

### 2.19 Statistical analysis

Statistical analyses were performed with graphpad 6.0 software (La Jolla, CA, USA). Tumor volumes were analyzed by Kruskal–Wallis nonparametric test with pairwise Wilcoxon test correction for multiple comparison. We used one‐way ANOVA with Tukey's multiple comparison test for comparing more than two groups. Unpaired *T*‐test analysis with Welch's correction was used when two groups were compared. Significance was considered when *P* < 0.05.

## 3 Results

### 3.1 Deregulation of citrate metabolism increases gemcitabine sensitivity

**Fig. 1:**
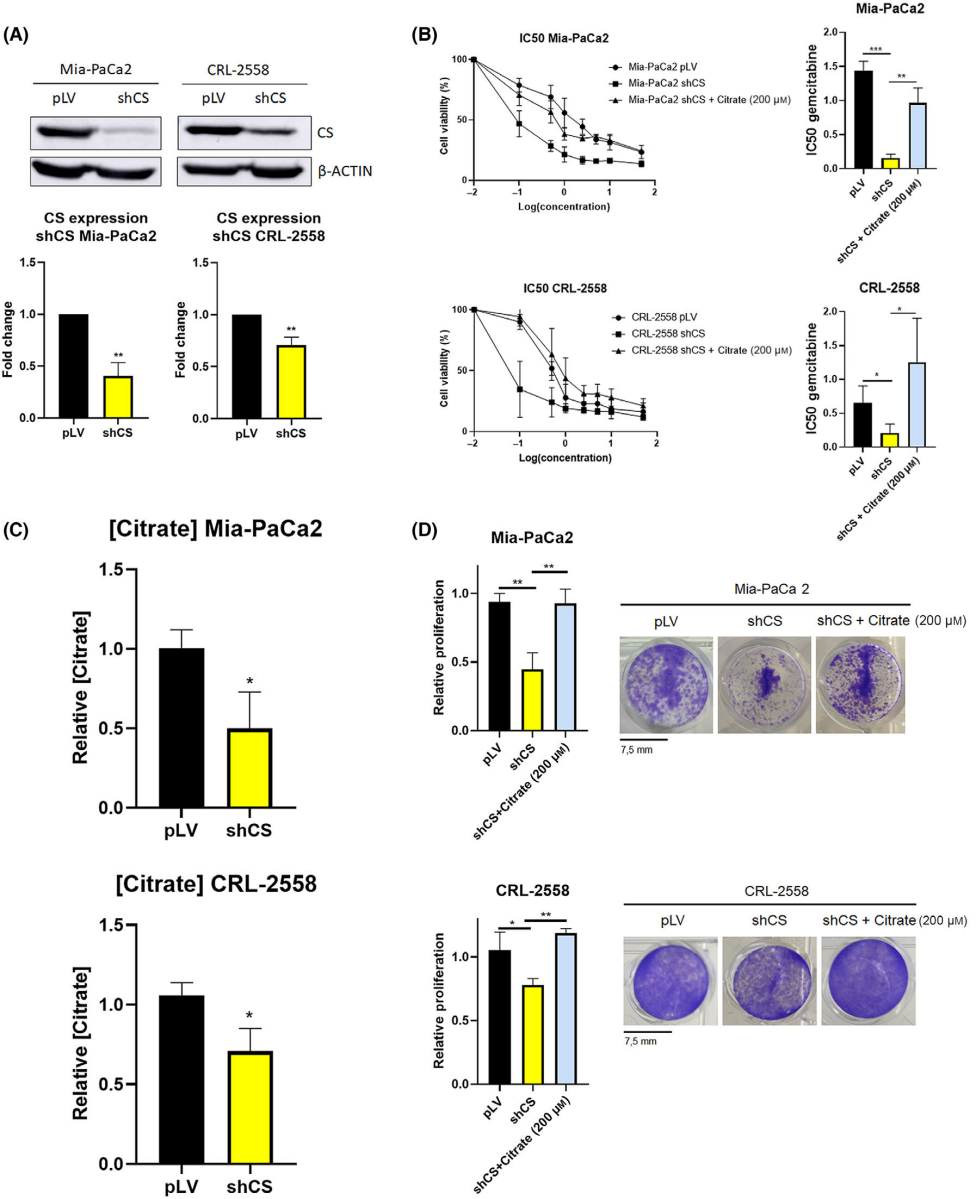
Citrate Synthase (CS) knockdown sensitizes pancreatic ductal adenocarcinoma cell lines to gemcitabine. (A) Western Blot showing CS expression in empty vector (pLV) and pLV CSshRNA transfected Mia‐PaCa2 and CRL‐2558 cell lines (*n* = 3). (B) IC50 of gemcitabine in pLV empty vector, shCS and shCS +200 μm citrate (*n* = 3). (C) Relative citrate concentration in pLV empty and pLV CSshRNA transfected Mia‐PaCa2 and CRL‐2558 cell lines (*n* = 3). (D) Proliferation assay of pLV empty, pLV CSshRNA and pLV CSshRNA +200 μm citrate transfected Mia‐PaCa2 and CRL‐2558 cell lines (*n* = 3). Data are presented as mean ± SD. Statistical analyses were determined by two‐tailed unpaired *t*‐test (*P < 0.05; **P < 0.01) in case of panel A & C, and using one‐way ANOVA with Tukey's multiple comparison test (*P < 0.05; **P < 0,01; ***P < 0.001) in B & D panels.

Excessive Citrate Synthase (CS) activity has previously been observed in pancreatic tumor tissue when compared to adjacent non‐neoplastic tissue to feed tumor with citrate [8]. However, existing literature has not established any connection between this heightened CS activity and overall survival or treatment response in pancreatic cancer. In this study, we present evidence demonstrating that the knockdown of CS in Mia‐Paca 2 and CRL‐2558 cell lines (Fig. 1A) results in increased sensitivity to gemcitabine, a commonly used chemotherapy drug for pancreatic cancer (Fig. 1B). This heightened sensitivity was particularly pronounced in the Mia‐Paca 2 cell line where CS levels were reduced by nearly 70%. In the CRL‐2558 cell line, where CS silencing was achieved at approximately 40%, a similar but slightly less pronounced reduction in gemcitabine's IC50 was observed (Fig. 1B). The enhanced sensitivity could be attributed to a significant reduction in citrate levels following CS knockdown (Fig. 1C). Proliferation assays, depicted in Fig. 1D, revealed a notable decrease in the propagation of both cell lines when CS was knocked down. Interestingly, when we reintroduced physiological concentrations of citrate (200 μm), the silenced phenotype of the cells was completely rescued. This citrate supplementation led to an increase in the proliferation of the silenced cell lines (Fig. 1D) and a corresponding enhancement in gemcitabine's IC50 (Fig. 1B). The same effect was observed when citrate catabolism was altered. Knockdown of FASN in Mia‐Paca2 and CRL‐2558 cell lines resulted to be synergetic with gemcitabine. Higher apoptotic rates were observed upon gemcitabine treatment when FASN protein levels were knockdown (Fig. S1). These findings strongly suggest that citrate availability and metabolism plays a crucial role in determining both the proliferative capacity of the cells and their sensitivity to gemcitabine.

### 3.2 Ascorbic acid inhibits citrate synthase reducing glucose‐derived citrate levels in PDAC

Citrate Synthase is a key enzyme in pancreatic cancer metabolism. Apart from CS being necessary for correct TCA cycle function, citrate is the substrate for the majority of newly synthetized lipids in pancreatic cancer [23]. In this context, we aimed to check out for putative changes in Citrate Synthase after vitamin C and gemcitabine treatments.

**Fig. 2:**
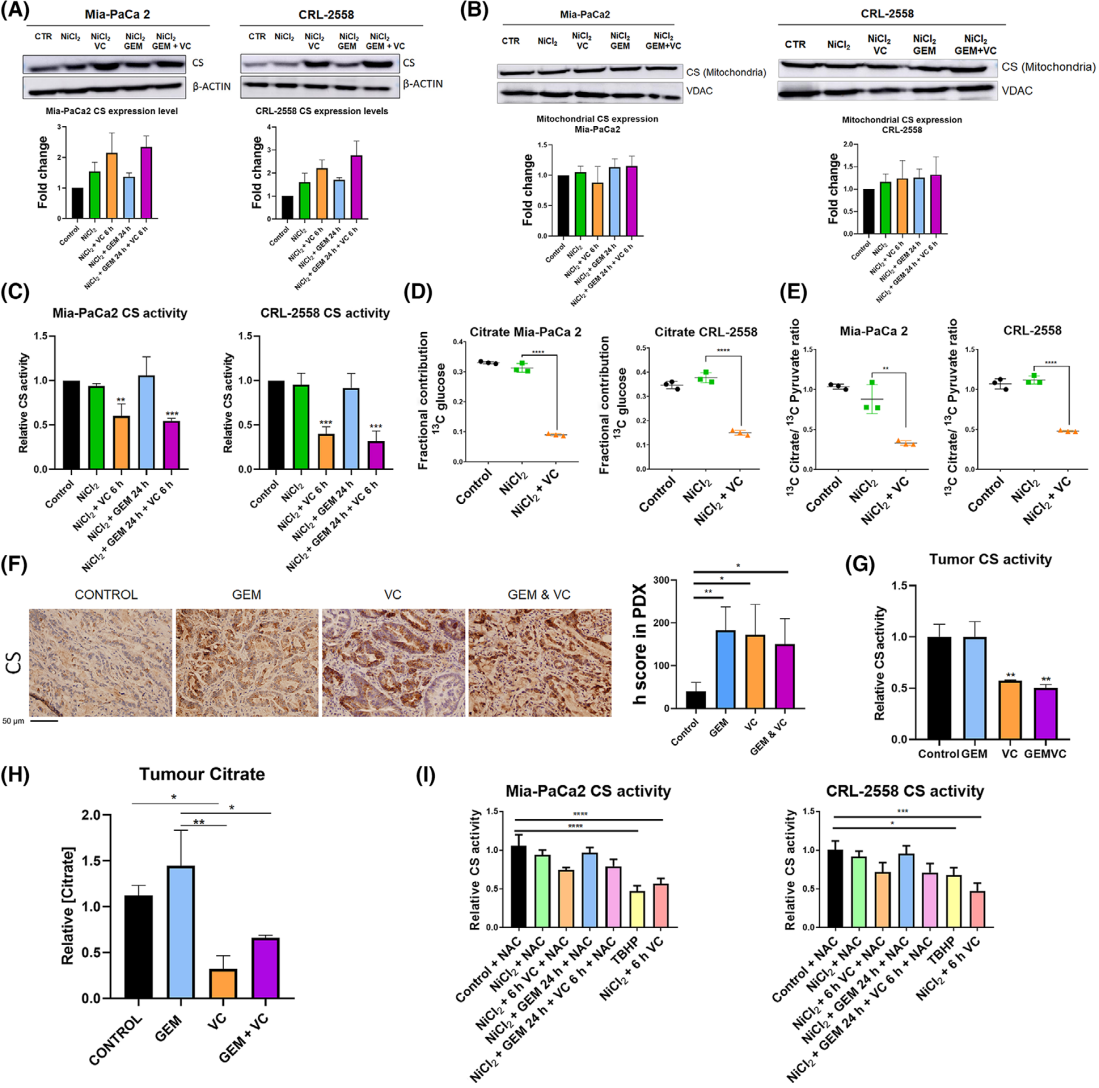
Vitamin C inhibits citrate synthase activity. (A) Citrate synthase (CS) protein expression in Mia‐PaCa 2 and CRL‐2558 cell lines after ascorbic acid, gemcitabine or combinatory treatment (*n* = 3). (B) mitochondrial CS expression in Mia‐Paca 2 and CRL‐2558 cell lines upon ascorbic acid, gemcitabine or combinatory treatment (*n* = 3). (C) CS activity in both pancreatic ductal adenocarcinoma cell lines after ascorbic acid, gemcitabine or combinatory treatment (*n* = 3). (D) 13C Glucose conversion into citrate in Mia‐Paca 2 and CRL‐2558 in hypoxic conditions and after vitamin C treatment. Data is represented as fractional contribution ± SD. All experiments were set in triplicate. (E) Ratio between ^13^C glucose‐derived citrate and pyruvate in both PDAC cell lines upon Nickel Chloride (NiCl2) and vitamin C exposure for 4 h (*n* = 3). (F) CS expression in mice tumors treated with vitamin C (VC), Gemcitabine (GEM), or combinatory treatment (*n* = 5) Scale Bar Measurement = 50 μm. (G) CS activity in PDAC mice tumors treated with vitamin C, Gemcitabine (GEM) or combinatory treatment (*n* = 5). (H) Citrate levels in PDAC Patient‐derived xenograft (PDX) after vitamin C, gemcitabine or combinatory treatment (*n* = 5). (I) Relative CS activity in Mia‐PaCa2 and CRL‐2558 cell lines upon N‐Acetyl Cystein (NAC) pretreatment and exposure of cells to NiCl_2_, vitamin C and/or gemcitabine. Data are presented as mean ± SD (unless specified) Statistical analyses were determined by one‐way ANOVA with Tukey's multiple comparison test (*P < 0.05; **P < 0.01; ***P < 0.001; ****P < 0.0001).

Strikingly, WB analysis showed a moderate upregulation of CS expression when PDAC cell lines were treated with vitamin C and combinatory treatment (Fig. 2A). Gemcitabine alone just slightly enhanced CS protein levels. Nevertheless, mitochondrial CS levels remained stable upon both treatments (Fig. 2B). Overall VDAC1 expression was increased after vitamin C treatment (Fig. S2), which suggested mitochondrial pool to be increasing upon ascorbate exposure. Surprisingly, when the activity of the CS was checked, a clear and severe inhibition was observed upon vitamin C in monotherapy or gemcitabine plus vitamin C exposure (Fig. 2C). 13CGlucose tracing experiments validated the above‐mentioned experiments. Glucose‐derived labeled citrate was significantly reduced upon vitamin C treatment. M2 citrate levels were lowered when cells were treated with vitamin C, validating less citrate is being generated from CS in cells treated with ascorbate (Fig. 2D). Additionally, we examined the ratio between labeled citrate and pyruvate as a potential indicator of CS activity. In the presence of vitamin C, this ratio was significantly reduced, further supporting the notion that CS activity is diminished under vitamin C treatment conditions (Fig. 2E). PDX tumor analysis revealed exactly the very same results. CS protein expression was significantly enhanced in gemcitabine, vitamin C and combinatory treatments (Fig. 2F) while the activity was lowered just in vitamin C conditions (Fig. 2G). All activity assays were normalized to protein expression. Furthermore, our investigation revealed that tumor citrate levels were notably reduced when exposed to vitamin C treatment conditions, as illustrated in Fig. 2H. This finding underscores the impact of vitamin C on citrate synthase activity in the context of tumor metabolism.

To elucidate a possible mechanism behind this intriguing inhibition, cells were treated with N‐acetylcysteine. Addition of N‐acetylcysteine to the culture media as a ROS inhibitor resulted to block the inhibitory effect produced by vitamin C. Citrate synthase was barely inhibited by ascorbic acid upon NAC treatment, suggesting a ROS‐dependent inhibition of vitamin C for CS. Moreover, the ROS inductor TBHP also reduced CS activity, confirming possible ROS dependent inhibition of the enzyme (Fig. 2I).

### 3.3 Vitamin C downregulates key citrate metabolic enzymes, ACLY and FASN

Human ATP citrate lyase (ACLY) and fatty acid synthase (FASN) are fundamental multi‐enzymatic activity proteins for citrate to be metabolized in cancer cells. They are involved in citrate‐dependent *de novo* lipogenesis, a major metabolic process, converting glucose into palmitate, the building block for more complex fatty acids for proliferation. Overexpression of these enzymes has been reported in pancreatic ductal adenocarcinoma which increased chemoresistance to gemcitabine [23].

**Fig. 3:**
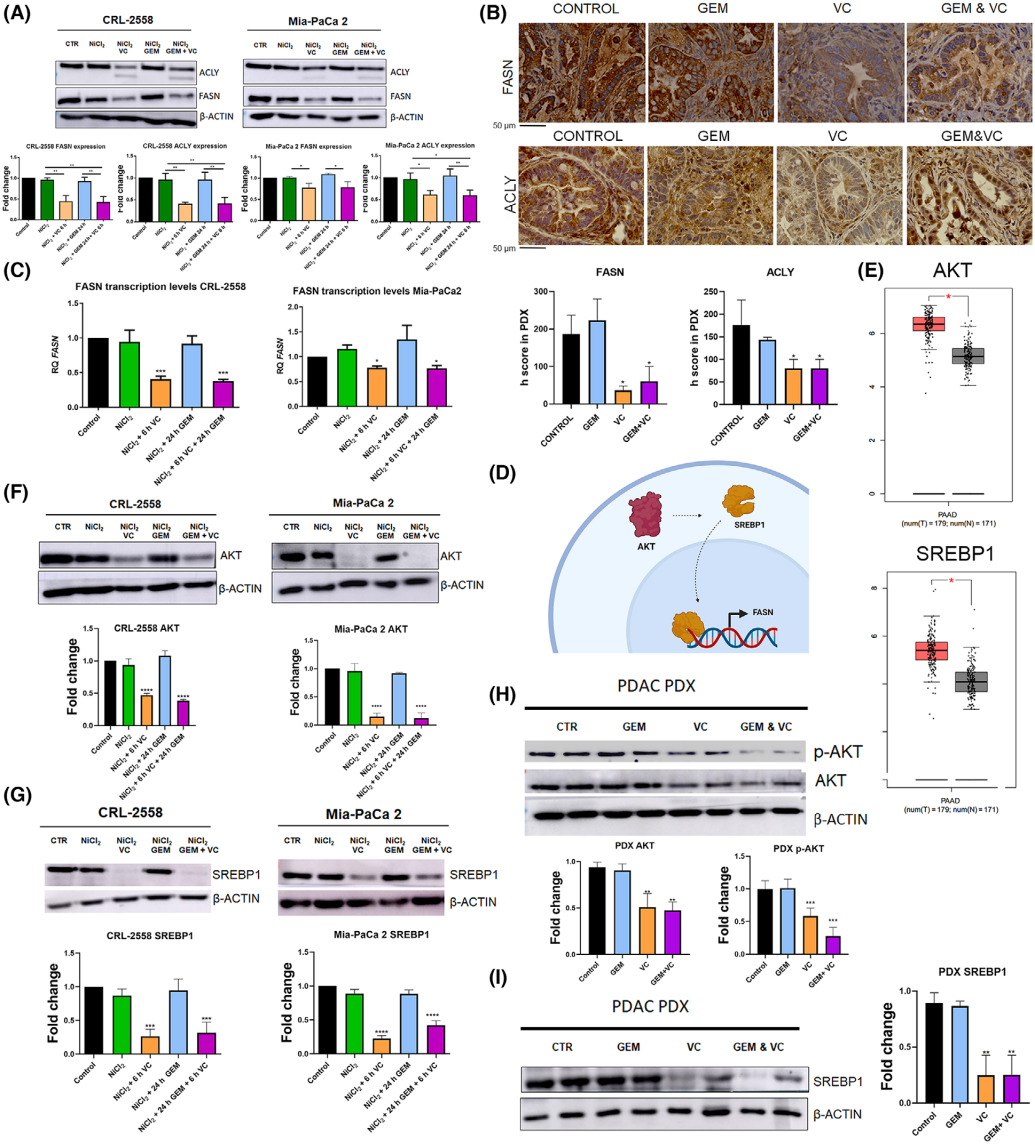
Ascorbic acid‐dependent downregulation of Fatty Acid Synthase (FASN) and ATP Citrate Lyase (ACLY) through AKT/SREBP1 axis modulation. (A) FASN and ACLY protein expression after NiCl_2_ (Nickel chloride), ascorbate, gemcitabine or combinatory treatment in Mia‐PaCa2 and CRL‐2558 cell lines (*n* = 3). (B) Immunohistochemistry analysis and h score bars showing FASN and ACLY expression in mice tumors treated with Gemcitabine (GEM), vitamin C or combinatory therapy (*n* = 5) Scale Bar Measurement = 50 μm. (C) *FASN* gene expression in Mia‐Paca2 and CRL‐2558 after NiCl_2_, GEM, vitamin C or combinatory treatment (*n* = 3). (D) Schematic representation of FASN transcriptional regulation through AKT. (E) The Cancer Genome Atlas (TGCA database) analysis of pancreatic tumor (red) versus healthy tissue (gray) of *AKT* and *SREBP1* expression. F and G AKT and SREBP1 protein expression after NiCl_2_, vitamin C, GEM or combinatory treatment in both PDAC cell lines (*n* = 3). (H, I) AKT, p‐AKT (Ser 473) and SREBP1 tumor expression (*n* = 5) Data are presented as mean ± SD. Statistical analyses were done using one‐way ANOVA with Tukey's multiple comparison test (*P < 0.05; **P < 0.01; ***P < 0.001; ****P < 0.0001).

We aimed to check out whether FASN and ACLY might be regulated by vitamin C in our *in vitro* and patient‐derived xenograft (PDX) models. WB analysis revealed that vitamin C was able to downregulate FASN and ACLY expression (Fig. 3A) in CRL‐2558 and Mia‐PaCa2 human pancreatic cancer cell lines. 3 mm ascorbic acid exposure for 6 h significantly lowered FASN and ACLY protein levels alone or in combination with gemcitabine. Gemcitabine treatment did not affect FASN nor ACLY protein expression when used as monotherapy.

As pancreatic cancer is known to be a highly hypoxic tumor, NiCl_2_ was added to cells in order to stabilize HIF1α and mimic original tumor conditions (Fig. S3).

FASN and ACLY immunohistochemistry tumor staining of PDX samples validated what it was previously observed *in vitro*. Tumors treated with pharmacological doses of vitamin C (4 g·kg^−1^) for 15 days or combination of gemcitabine (30 mg·kg^−1^) plus ascorbic acid drastically reduced FASN levels compared with the ones treated with vehicle or gemcitabine (Fig. 3B). ACLY expression was also moderately reduced in vitamin C‐treated tumors (Fig. 3B).

qPCR experiments demonstrated transcriptional regulation of FASN when Mia‐PaCa2 and CRL‐2558 cell lines were treated with vitamin C in combinatory treatment. FASN transcript levels were lowered (specially in CRL‐2558) to a half after 6 h of vitamin C exposure, while levels were maintained when the same cells were treated with gemcitabine alone (Fig. 3C).

As AKT/SREBP1 axis is responsible for FASN expression [24] (Fig. 3D) and both protein are upregulated in PDAC (Fig. 3E), we decided to check the levels of these two proteins in our *in vitro* model. Ascorbic acid treatment dramatically downregulated AKT expression in Mia‐PaCa2 cell line, while more moderate but significant downregulation was observed in CRL‐2558 cell line (Fig. 3F). The same results were observed with SREBP1 transcription factor. SREBP1 protein levels were significantly lowered when vitamin C was added to the cells for 6 h (Fig. 3G). Again, NiCl_2_ was added to the culture media to mimic tumor conditions.

Preclinical PDX model validated the previous results. Tumors treated with vitamin C or combinatory treatment of vitamin C plus gemcitabine presented lower levels of AKT (Fig. 3H) and SREBP1 protein levels (Fig. 3I), strongly suggesting a vitamin C‐dependent downregulation of FASN and ACLY transcription through AKT/SREBP1 axis. p‐AKT levels were also reduced upon vitamin C treated tumors in concordance of AKT reduction (Fig. 3H).

### 3.4 Deregulated citrate metabolism correlates with reduced LDHA levels after vitamin C treatment

FASN downregulation is known to be correlated with reduced aerobic glycolysis and increased lactate production [24]. Moreover, TGCA analysis revealed strong correlation between citrate metabolism implicated enzymes and main Warburg metabolism mediators in pancreas (Fig. S4A). LDHA is responsible of converting pyruvate to lactate and then regenerating NAD^+^, in order to sustain the glycolytic pathway. The overexpression of LDHA in pancreatic cancer is also associated with poor prognosis and resistance to standard chemotherapeutic regimes [25].

We aimed to test LDHA levels in both PDAC cell lines after vitamin C treatment.

**Fig. 4:**
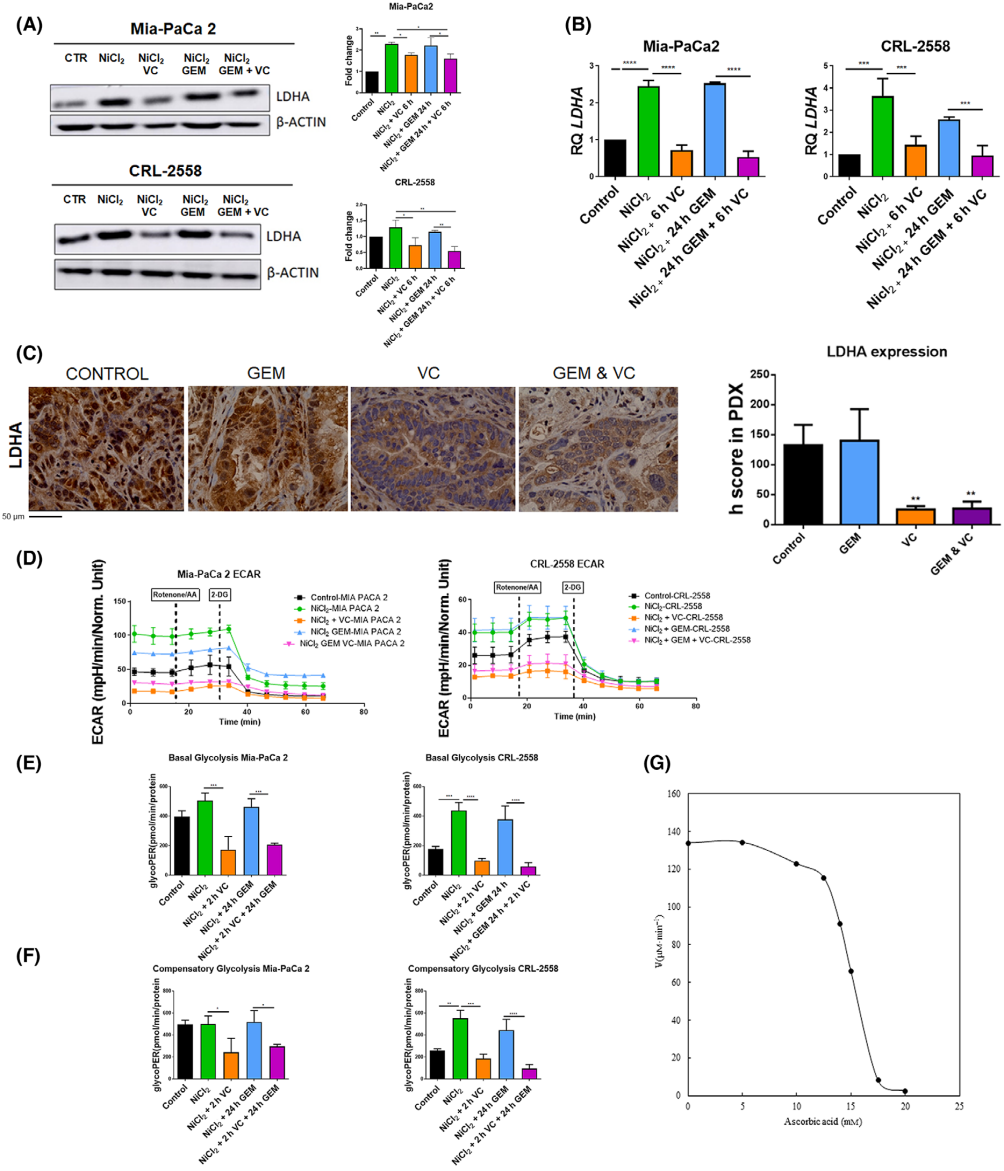
Vitamin C‐dependent downregulation of Lactate Dehydrogenase A (LDHA). (A, B) LDHA protein and gene expression in pancreatic cancer (PDAC) cell lines treated with NiCl_2_ (Nickel Chloride), vitamin C, Gemcitabine (GEM) or combination of both treatments. Protein expression was normalized to β‐Actin and gene expression to *RPLP0* (*n* = 3). (C) LDHA *in vivo* protein expression in mice tumors in response to vitamin C, GEM or combination (*n* = 5) Scale Bar Measurement = 50 μm. (D–F) ECAR (Extracellular acidification rate), basal glycolysis and compensatory glycolysis in Mia‐PaCa2 and CRL‐2558 after NiCl_2_, ascorbic acid, gemcitabine or combination (*n* = 5). GlycoPER = Glycolutic Proton Efflux Rate; AA = Antimycin A; 2‐DG = 2‐Deoxy‐D‐glucose. (G) Effect of vitamin C concentration on the LDHA activity. The reaction medium at 25 °C contained 100 mm sodium phosphate buffer pH 7.0, 250 μm pyruvate, 10 μm NADH, 1 mg·mL^−1^ LDHA and increasing concentrations of ascorbic acid (0–20 mm) (*n* = 3). Data are presented as mean ± SD. Statistical analyses were done using one‐way ANOVA with Tukey's multiple comparison test (*P < 0.05; **P < 0.01; ***P < 0.001; ****P < 0.0001).

WB analysis showed moderate downregulation of LDHA protein expression in vitamin C added conditions (Fig. 4A). Interestingly, NiCl_2_ slightly upregulated its expression, suggesting hypoxic‐dependent upregulation of LDHA in PDAC. However, vitamin C alone is still able to downregulate LDHA expression (Fig. S4B,C).

Transcriptional analysis by qPCR yielded results supporting those observed by WB. Hypoxia‐upregulated LDHA transcription upon vitamin C addition decreased LDHA transcription levels (Fig. 4B). Gemcitabine monotherapy did not affect LDHA protein nor transcription levels, suggesting all reduction observed was probably produced by ascorbic acid treatment. PDX tumor analysis displayed also an extremely downregulation of LDHA in vitamin C treated tumors (Fig. 4C).

As lactate production is one of the major cause of extracellular acidification in PDAC tumors [26], extracellular acidification rate (ECAR) was measured after vitamin C exposure in PDAC Mia‐PaCa2 and CRL‐2558 cell lines. As expected, addition of NiCl_2_ (to mimic hypoxic conditions) enhanced acidification rates; while ascorbic acid dramatically reduced ECAR levels in both cell lines (Fig. 4D). Moreover, both basal glycolysis and compensation glycolysis were clearly lowered upon vitamin C treatment (Fig. 4E,F).

Apart from LDHA being downregulated, kinetic analysis of pure LDHA revealed direct inhibitory effect of vitamin C in the enzymatic activity. As observed with CS, LDHA activity was impaired by direct addition of ascorbic acid to the reaction medium, reaching complete inhibitory effect in the presence of 20 mm of ascorbic acid (Fig. 4G).

### 3.5 Vitamin C downregulates GLUT1 and PDK1, enhancing PDH activity

Effect of vitamin C on LDHA expression, activity and lactate concentration in PDAC points out to an important role of ascorbic acid on aerobic glycolysis. These data encouraged us to study this metabolic pathway in more depth.

GLUT‐1 is a main glucose transporter and it has been described as a predictor of worse prognosis in pancreatic adenocarcinoma regulating the tumor immune microenvironment and also promoting tumor metastasis in PDAC [27].

Pyruvate dehydrogenase kinases (PDK) are key enzymes which increase the flux of pyruvate into the mitochondria, promoting glucose oxidation over glycolysis through the inactivation of pyruvate dehydrogenase (PDH).

PDK1 is dominantly found in pancreatic islets and it has been described that inhibition of PDK1 influence microbiota and metabolomic profile in pancreatic cancer xenograft mice. Inhibition of PDKs has been suggested as a promising therapeutic option in pancreatic cancer [28].

**Fig. 5:**
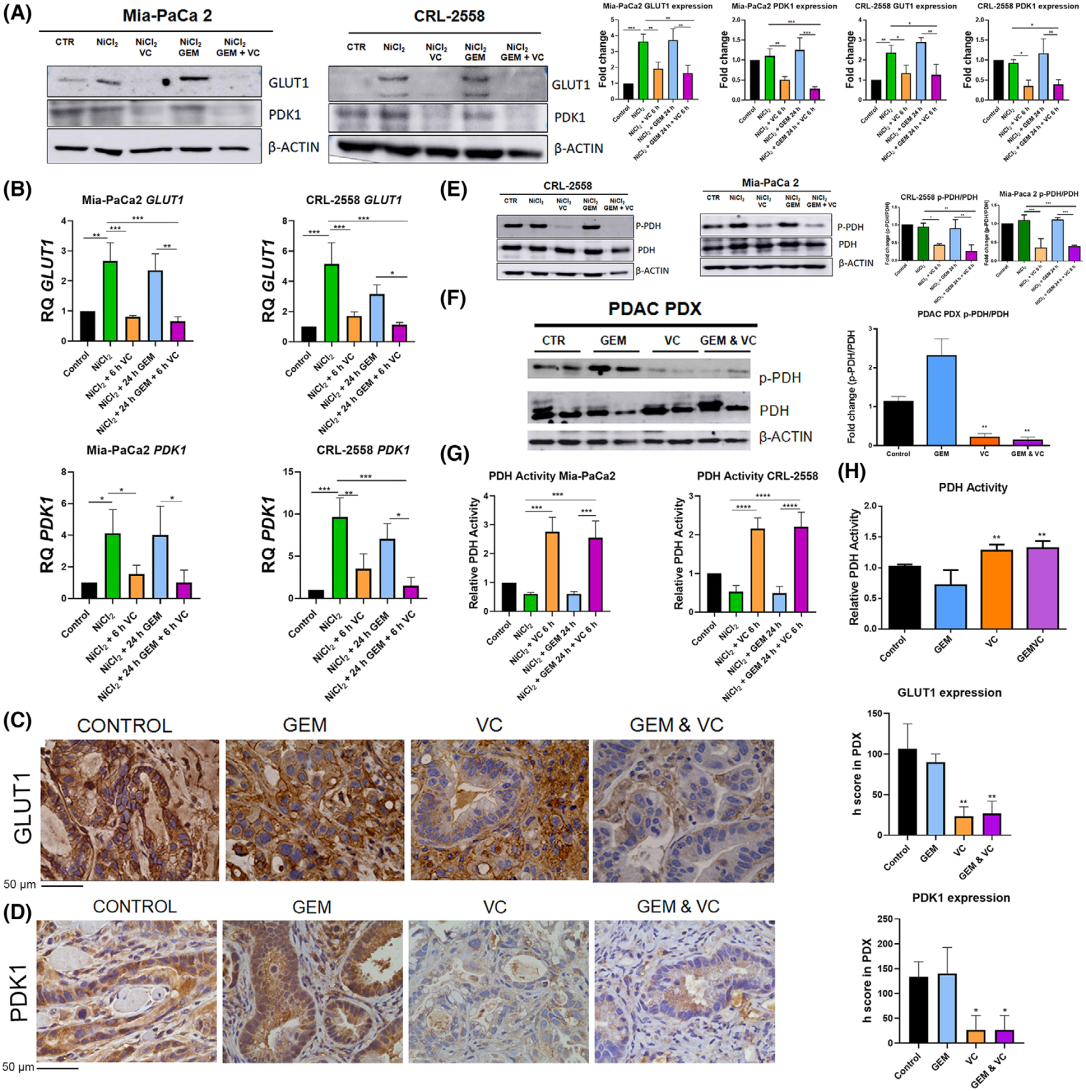
Vitamin C targets GLUT1 and PDK1, enhancing PDH activity. (A, B) GLUT1 and PDK1 protein and gene expression in response to NiCl_2_, vitamin C, gemcitabine or combination in Mia‐PaCa2 and CRL‐2558 cell lines (*n* = 3). (C, D) Immunohistochemical analysis and its h score bars of PDAC PDX tumors showing GLUT1 and PDK1 expression in mice treated with vitamin C, Gemcitabine GEM or combinatory treatment (*n* = 5). Scale bars = 50 μm. (E) Relative p‐PDH/PDH protein expression in PDAC cell lines (*n* = 3) after NiCl_2_, vitamin C, gemcitabine or combination treatment. (F) Relative p‐PDH/PDH protein expression in Patient‐derived xenograft tumors (PDX) (*n* = 5) after vitamin C, gemcitabine or combination treatment. (G) PDH activity in PDAC cell lines (*n* = 3). (H) PDH activity in PDX tumors (*n* = 5) in response to vitamin C, gemcitabine or combination. Data are presented as mean ± SD. Statistical analyses were determined by one‐way ANOVA with Tukey's multiple comparison test (*P < 0.05; **P < 0.01; ***P < 0.001; ****P < 0.0001).

GLUT1 and PDK1 expression were then evaluated. Protein and mRNA modulation of both, GLUT‐1 and PDK1 in Mia‐PaCa2 and CRL‐2558 cell lines was observed when cells were treated with 3 mm ascorbic acid for 6 h (Fig. 5A,B). Vitamin C significantly downregulated GLUT1 and PDK1 levels at these conditions. Mimicking hypoxia environment with NiCl_2_ significantly upregulated the transcriptional expression of these two proteins, strongly suggesting a HIF1α dependent transcription of GLUT1 and PDK1 in PDAC. In normoxia, vitamin C alone was still able to downregulate PDK1 levels (Fig. S4B,C).

GLUT1 membrane staining as well as PDK1 score were downregulated in tumors treated with vitamin C or combinatory treatment of vitamin C and gemcitabine (Fig. 5C,D).

PDK1 downregulation with vitamin C correlated with reduced phosphorylation of Ser239 in pyruvate dehydrogenase in both cell lines (Fig. 5E). *In vivo* model showed very similar results, although phosphorylated PDH expression was enhanced in tumor treated with gemcitabine (Fig. 5F).

Interestingly, enzyme activity assays performed revealed that PDH activity was 2–3 times higher in cells treated with ascorbic acid, suggesting a switch in metabolism (Fig. 5G). Tumor analysis confirmed the stated above. Tumor that received vitamin C turned to have enhanced activity of PDH (Fig. 5H).

### 3.6 Vitamin C‐induced PDAC cell lines apoptosis enhancing gemcitabine efficacy

**Fig. 6:**
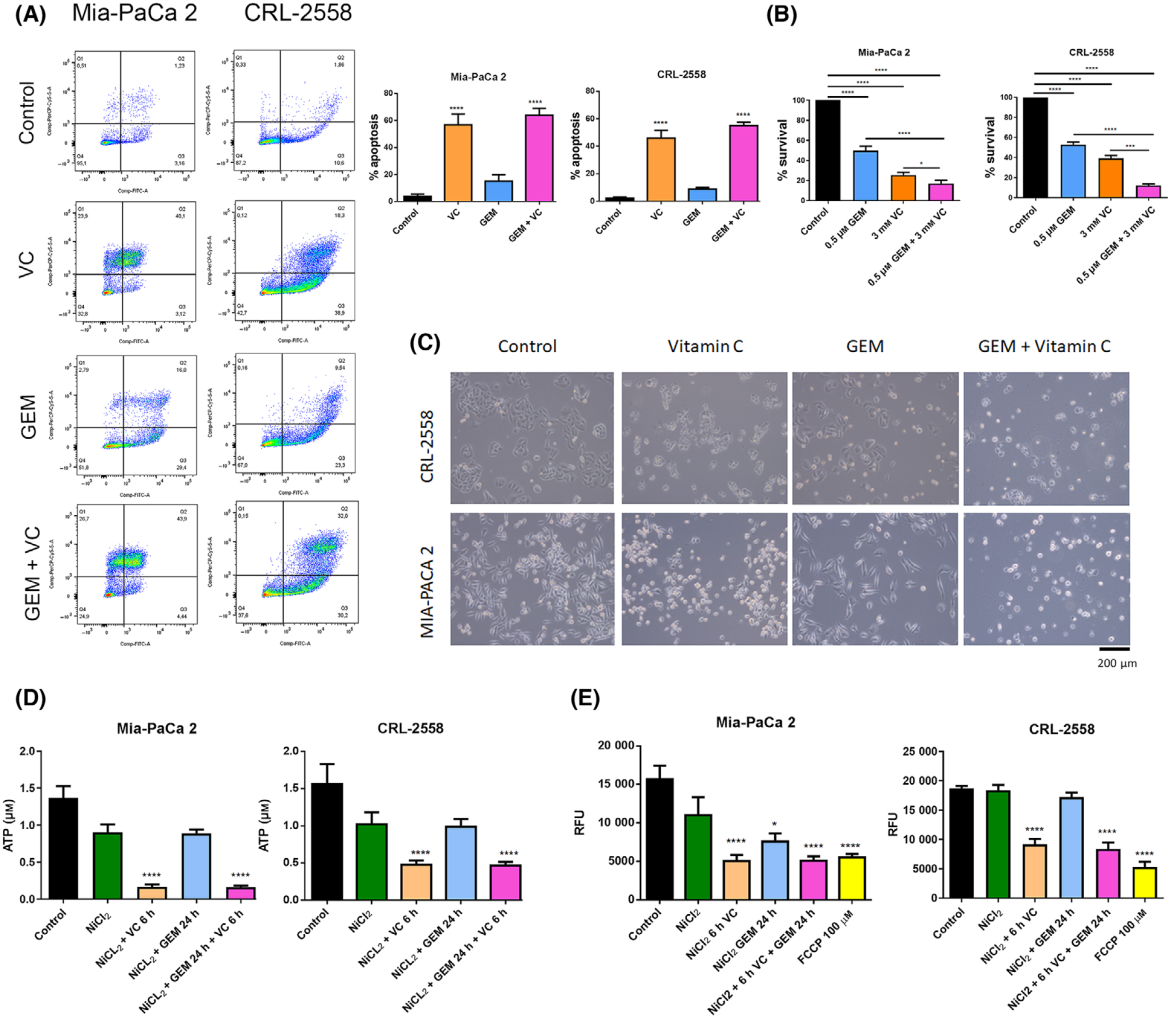
Vitamin C synergizes with Gemcitabine in pancreatic cancer cells inducing apoptosis. (A) Annexin V‐PI (Propidium Iodide) based apoptosis analysis in Mia‐PaCa2 and CRL‐2558 cell lines in response to vitamin C, gemcitabine or combinatory treatment (*n* = 3). (B) Viability assay of PDAC cell lines after, vitamin C, Gemcitabibe (GEM) or combination (*n* = 3). (C) Representative images showing cell morphology after vitamin C, GEM or combinatory treatment (*n* = 3) Scale bar = 200 μm. (D, E) ATP production and mitochondrial membrane potential in Mia‐PaCa2 and CRL‐2558 cell lines after vitamin C, gemcitabine or combination (*n* = 3). FCCP was used as a positive control for mitochondrial potential loss. RFU, relative fluorescence value. Data are presented as mean ± SD. Statistical analyses were done using one‐way ANOVA with Tukey's multiple comparison test (*P < 0.05; ***P < 0.001; ****P < 0.0001).

To check if combinatory treatment of vitamin C and gemcitabine might have synergetic effects, apoptosis assay were performed. Annexin V‐Pi analysis revealed that, although not significant, higher apoptotic rates were achieved when Mia‐PaCa2 and CRL‐2558 cell lines were treated with gemcitabine plus ascorbic acid (Fig. 6A). 5–10% more apoptosis was observed in the combinatory conditions if they were compared with vitamin C treatment alone, showing a potential synergetic effect.

Viability assays performed counting viable cells after treatments revealed the same result. Combination of vitamin C and GEM resulted in clearly higher cell death (Fig. 6B), effect that is clearly depicted in optical microscope images (Fig. 6C). Furthermore, ATP production was drastically reduced when ascorbic acid was added to both PDAC cell lines (Fig. 6D). NiCl_2_ also reduces ATP level as glycolysis being induced ATP production is less effective. All these results correlated with a lowered mitochondrial membrane potential in vitamin C conditions (Fig. 6E), validating apoptosis assay results.

### 3.7 Vitamin C enhances gemcitabine response in a PDAC PDX model

Increased *de novo* lipogenesis in patients with pancreatic cancer is associated with greater resistance to therapeutic treatment with gemcitabine [10]. CS inhibition could be an option to increase the therapeutic effect of gemcitabine in this type of tumor with so few therapeutic options.

To validate that CS inhibition via vitamin C correlated with a higher response of gemcitabine *in vivo* a PDX model was established.

**Fig. 7:**
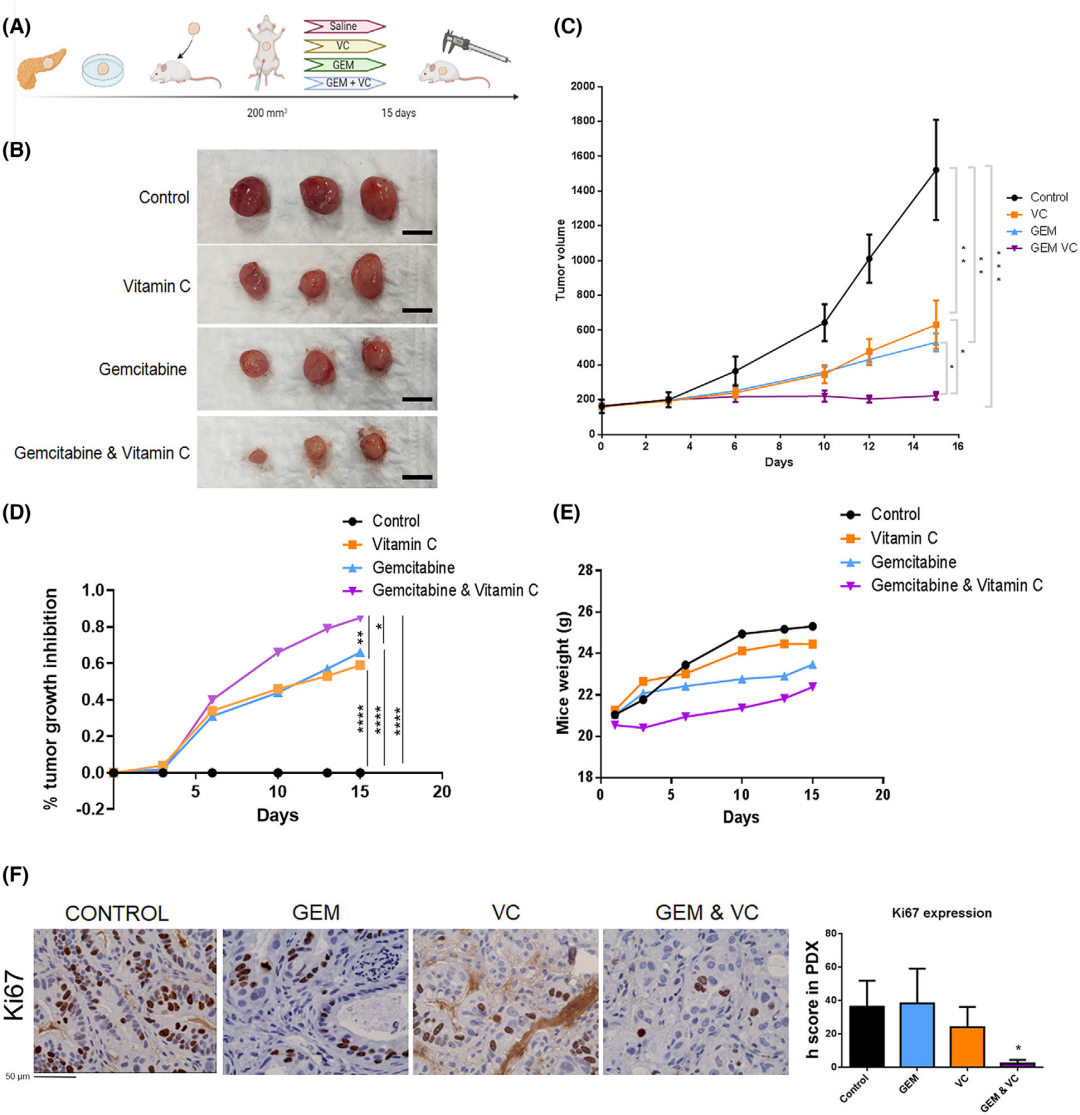
Vitamin C enhances gemcitabine response in PDAC PDX model. (A) Schematic representation of the PDX model treatment design. (B) Tumor images at the end of the treatment. (C) Tumor volume curve in response to saline, vitamin C, gemcitabine of combinatory treatment (*n* = 9 per group). (D) Percentage of tumor growth inhibition during mice treatment (*n* = 9 per group) Scale bars = 5 mm. (E) Mice weight during treatment infusion (*n* = 9 per group). (F) Ki67 protein expression in PDAC mice tumors after saline, vitamin C, gemcitabine or combination (*n* = 5) Scale Bar Measurement = 50 μm. Data are presented as mean ± SEM. Statistical analyses were determined by one‐way ANOVA with Tukey's multiple comparison test. Tumor volumes were analyzed by Kruskal–Wallis nonparametric test with pairwise Wilcoxon correction for multiple comparison (*P < 0.05; **P < 0.01; ***P < 0.001; ****P < 0.0001).

When subcutaneously implanted tumors reached 200 mm^3^, intraperitoneal treatment of mice started. Mice were sacrificed at day 16, when control group tumors reached 1500 mm^3^ of volume (Fig. 7A).

At this time, gemcitabine monotherapy reduced tumor volume to 50–60%, while 85% of tumor growth inhibition was achieved in the combinatory treatment between gemcitabine plus vitamin C (Fig. 7B,C). Notably, ascorbic acid alone presented similar results as gemcitabine monotherapy, reducing tumor volume around 50%.

But the most surprising result was obtained by combining gemcitabine with vitamin C, since the tumor volume experienced an almost negligible increase compared to monotherapy and not treated conditions. This observation clearly points out to a synergistic activity of both molecules (Fig. 7B,C). Significant weight loss in mice was not observed during treatment (Fig. 7D).

Ki67 staining revealed weak staining in the combinatory treatment tumor tissue (Fig. 7E). Surprisingly, although tumor volume had been affected, no significant Ki67 differences were observed in monotherapy treatments. Ki67 remained stable in both vitamin C or gemcitabine therapy alone.

## 4 Discussion

Metabolic reprogramming has been considered a hallmark in cancer since 2011 [29]. In the last years, lipid metabolism has raised as an important deregulated pathway in various neoplasias [30]. In 2017, Analysis from the Cancer Genome Atlas revealed lipid metabolism to be the most correlated pathway with poor gemcitabine response in PDAC [10].

Cancer cells need to switch their metabolism to meet their demands of rapid cell growth and proliferation [31]. The need of a constant supply of lipids and cholesterol for new membrane construction makes tumoral cells to shift their metabolism towards increased lipogenesis through mitochondrial citrate [32, 33]. Enhanced citrate metabolism towards lipid synthesis favors malignant phenotype in cancer.

Glucose is one the main sources of lipids in cancer cells. Citrate Synthase, a vital enzyme, serves as a crucial link between glucose and lipid metabolism. In the context of pancreatic cancer, approximately 93% of newly formed lipids are synthesized through mitochondrial citrate, placing citrate as a rate‐limiting substrate for tumorigenity [23]. Citrate metabolism plays a pivotal role in facilitating rapid cell proliferation and promoting tumor growth. Experimental studies have demonstrated that physiological citrate levels (200 Um) sustain proliferation of pancreatic cancer cells and that concentrations up to 5 Mm enhance *de novo* lipogenesis [34] Notably, individuals with pancreatic cancer exhibit diminished levels of citrate in plasma due to the high demand for citrate to be metabolized within the tumor [35]. Additionally, research has shown that cancer‐associated cells actively supply the tumor with citrate, thereby fostering the progression of tumor metastasis [36].

Enhanced activity of CS has been described in PDAC compared with adjacent non neoplasic tissue [8]. Normal healthy cells use CS for catabolic TCA cycle and energy production. Pancreatic cancer cells, instead, increase the activity of such enzyme to derive citrate towards phospholipids and new membranes generation for proliferation [36].

Citrate Synthase knockdown has been described to be synergetic with cisplatin in ovarian cancer [37]. Here we have reported that CS silencing (Fig. 1A) resulted to be synergetic with gemcitabine treatment in pancreatic cancer cell lines (Fig. 1B). IC50 for gemcitabine was significantly reduced upon CS silencing in both cell lines. Lentiviral knockdown of the enzyme resulted in reduced citrate levels (Fig. 1C), which decreased the ability of cells to proliferate (Fig. 1D). The phenotype was completely rescued when CS silenced cells were administered with physiological concentrations of citrate (Fig. 1B,D), placing citrate availability in pancreatic cancer as responsible for diminished gemcitabine sensitivity and proliferation.

Remarkably, vitamin C treatment in PDAC cells and PDX tumors resulted in an important impairing of the enzyme activity (Fig. 2C,G). 13C glucose tracing experiments validated CS activity to be inhibited when cells are treated with vitamin C (Fig. 2D,E). The activity of CS is majorly controlled by the availability of its substrates, acetyl‐CoA and oxalacetate [38]. It is well documented that vitamin C antitumoral effect relies on massive ROS production [39]. Elisabeth Schmidtmann et al. [40] reported in 2014 oxidation could reduce CS activity in Arabidopsis via formation of mixed disulfides. Addition of NAC to the culture media resulted in a major blockade of the inhibition, pointing out oxidative damage to be responsible for CS inhibition upon ascorbate treatment (Fig. 2I). NAC is able to reduce disulfide bridges. In this way, vitamin C treatment may induce disulfide bridges in citrate synthase, which significantly reduce the enzymatic activity. The mentioned CS inhibition led to reduced production of glucose‐derived citrate within the cells and tumor (Fig. 2D,H), which resulted in deregulation of citrate metabolism and diminished tumor proliferation.

Citrate serves as a substrate for enhanced lipogenesis which enriches cell membrane in saturated or mono unsaturated lipids, while the percentage of polyunsaturated lipids is reduced. Saturated lipids pack more densely, lowering the membrane hydrophilicity, which impairs lateral and transverse membrane dynamics. This process limits the uptake of polar and hydrophilic chemotherapeutic molecules into the cell, causing resistance [41]. Altered cell membrane composition also impairs cellular AKT signaling transduction [42, 43] and motility [44].

ATP citrate lyase (ACLY) and Fatty acid synthase (FASN) are key regulatory enzymes in citrate metabolism through *de novo* lipid synthesis pathway. Upregulation of these proteins has been described in multiple cancer types [45], including PDAC (Fig. S1A), correlating with poor prognosis [10, 46].

Increased FASN levels have been associated with resistance to gemcitabine in pancreatic cancer. FASN uses citrate‐derived manolyl‐Coa to fuel cancer cells with palmitate, which is converted into phospholipids that change cell membrane composition and polarity, then possibly altering the drug intake into the cell [47]. Reduced citrate metabolism through ACLY and FASN downregulation may correlate with altered lipid composition in vitamin C‐treated conditions, which helps the tumor to be more sensitive to gemcitabine. We have demonstrated that Ascorbic acid treatment regulates AKT/SREBP1 axis, downregulating key lipogenesis enzymes such as FASN and ACLY (Fig. 3A,B). Lower expression levels of FASN and ACLY may reduce citrate catabolism and generate a massive deregulation of *de novo* lipogenesis pathway, increasing gemcitabine uptake and improving tumor response.

In this study we report that vitamin C, a biomolecule essential for life and highly present in nature, plays a key role modulating the synthesis pathway of new lipids in PDAC by altering both citrate production and metabolism, and switching the hydrophilicity and permeability of the cancer cell membrane.

Several crucial citrate metabolic enzymes are transcriptionally regulated by SREBP1, including FASN and ACLY [48]. Oncogenic KRAS, which is present in almost 100% of PDAC patients, upregulates SREBP1 through PI3K/AKT/mTOR activation [49], thus increasing FASN and ACLY transcription in PDAC. Increased SREBP1 and AKT levels are observed in PDAC tumor tissue compared with their respective healthy tissue (Fig. 3E). Moreover, SREBP1 knockout is known to reduce tumor growth downregulating lipid generation in NSCLC [50]. PI3K/AKT/mTOR pathway regulation by ascorbic acid has been also described in prostate cancer [51] and it has been reported that vitamin C is able to induce AKT ubiquitination and degradation in thyroid cancer [52]. In this way, vitamin C is able to downregulate AKT expression in our model (Fig. 3F,H), which makes SREBP1 levels to decrease (Fig. 3G,I) and as a consequence, reduced FASN and ACLY protein levels are observed (Fig. 3A,B), altering the citrate metabolism towards the synthesis of new lipids.

Recently a non‐canonical function of SREBP1 has been described. SREBP1 knockdown resulted in reduced glycolytic levels and impaired oxidative phosphorylation [53]. FASN inhibition has also been associated with reduced glucose uptake and lactate production in breast cancer [54]. There is also a positive loop between FASN and AKT, as FASN inhibition by TVB‐3166 has demonstrated to downregulate PI3K‐AKT–mTOR axis reducing tumor growth in *Kras* mutant NSCLC mice [55]. Moreover, TGCA analysis of citrate metabolism signatures demonstrated strong correlation with main Warburg effectors in pancreatic cancer (Fig. S2). Akt is known to regulate key aerobic glycolysis enzymes in cancer enhancing Warburg effect [56]. Vitamin C‐dependent altered citrate metabolism (Fig. 3A,B) through AKT and SREBP1 downregulation (Fig. 3F–I) correlates with decreased GLUT1, PDK1 and LDHA (Figs 4A,B and 5A–D) levels in our model, suggesting a decreased glycolytic capacity in vitamin C‐treated tumors. Extracellular acidification rate analysis validated vitamin C ability to reduce both basal and compensatory glycolysis levels (Fig. 4D–F).

Hypoxic, NiCl_2_‐dependent GLUT1, PDK1 and LDHA upregulation (Figs 4B and 5B,C), strongly suggested a HIF1α‐dependent transcription of these glycolytic enzymes in PDAC. Pancreatic cancer is known to be a hypoxic tumor, where HIF1a is overexpressed [57]. In our previous study [16], we determined vitamin C was able to induce HIF1a hydroxylation and degradation, downregulating GLUT1 and PDK1 in KRAS mutant colorectal cancer. AKT is known to induce glycolysis through HIF1α induction in pancreatic cancer [58]. In this line, AKT downregulation (Fig. 3F,H) may correlate with HIF1α reduction by vitamin C and as a consequence in reduced transcription of glycolytic enzymes mentioned. PDK1 downregulation derived in reduced Ser293 phosphorylation of PDH and, in turn, in an enhanced enzyme activity, suggesting a switch in metabolism. Phosphorylation of Ser293 by PDKs is the main mechanism used in mammalians for PDH inactivation in cancer [59]. Activation of OXPHOS by PDK inhibitors have shown synergetic effects with standard chemotherapeutic agents in several tumors [60, 61].

Apart from citric acid metabolism, LDHA expression has also been associated with gemcitabine resistance in pancreatic cancer [62]. It has been reported that LDHA upregulation in PDAC promotes tumorigenesis [63]. Synergetic interactions have been described between LDHA inhibitors and gemcitabine treatment in hypoxic pancreatic cancer [64].

According to the results presented above, vitamin C‐treated tumors show a drastic reduction in LDHA protein levels (Fig. 4C). Moreover, *in vitro* kinetic experiments show a possible vitamin C‐dependent inhibition of the enzyme activity, suggesting another way of synergy between vitamin C and gemcitabine (Fig. 4G). Moreover, lactate is responsible for tumor microenvironment acidification [26], which reduces chemotherapeutics availability and intake into the tumor [65]. Lowering extracellular acidification by vitamin C (Fig. 4D) could increase gemcitabine uptake of pancreatic tumor cells, enhancing the drug action.

Furthermore, vitamin C role as a potential alkalizer of extracellular pH may be crucial to implement the use of other therapies, currently little used in pancreatic cancer due to their low efficacy.

Pancreatic ductal adenocarcinoma is generally characterized by an acidic tumor microenvironment (TME) that favors therapy resistance, cancer proliferation and immune evasion. Furthermore, recently it has been reported that functional inhibition of solute carrier family 4 member 4 (SLC4A4), the most abundant bicarbonate transporter expressed by epithelial ductal cells, improves T cell‐mediated immune response and impairs macrophage‐mediated immunosuppression, thus inhibiting tumor growth and metastases [66].

Vitamin C‐dependent inhibition of LDHA in PDAC might also decrease TME lactate concentration, thus lowering the acidic PDAC tumor microenvironment, favoring the action of T‐lymphocytes and opening the door to the use of novel biological immunotherapies. Interestingly, FASN overexpression has been also correlated with an immunosuppressive status in ovarian cancer, with a lower number and dysfunctional infiltrating T cells within the tumor [67]. Inhibition of FASN resulted in a partial rescue of immune response, which may suggest a potential role of vitamin C in boosting the efficacy of novel immunotherapies.

In this study we show that restriction of citrate availability and metabolism together with LDHA downregulation resulted in higher apoptotic rates in cells treated with gemcitabine plus vitamin C (Fig. 6A). These results correlate with the lowest ATP production and mitochondrial membrane potential levels observed in the combinatory treatment (Fig. 6D,E), suggesting the possible synergetic effect.

Surprisingly, an impressive 85% of tumor growth inhibition is described in the combinatory treatment of vitamin C and gemcitabine in our preclinical PDAC PDX model (Fig. 7B–D). Ascorbic acid enhances gemcitabine response increasing tumor growth inhibition around 25%. Tumors that received combinatory treatment barely proliferated. All this was confirmed with Ki67 immunohistochemistry staining (Fig. 7F), validating a potential synergetic effect between the two drugs in pancreatic cancer treatment. Published case reports have demonstrated objective pancreatic tumor regression with intravenous pharmacologic ascorbic acid therapy [68]. Moreover, phase I clinical trial performed in 2013 suggested some efficacy of pharmacological ascorbate plus gemcitabine infusion in pancreatic cancer patients [69], although the molecular mechanism ruling the synergistic effect between both molecules is still under scientific debate.

The overall data strongly suggest that the roles of mitochondrial Citrate Synthase in the production of cellular citrate and as a bridge between Warburg metabolism and the synthesis of new lipids have been underrated. The results here depicted, show this enzyme as an ideal candidate for the development of new antitumor strategies in the clinical management of pancreatic ductal adenocarcinoma, pointing out citrate metabolism as a target in pancreatin cancer.

## 5 Conclusions

This study describes non‐reported essential functions of vitamin C in pancreatic cancer (Graphical abstract). Ascorbic acid deregulates citrate metabolism by targeting CS and key enzyme involved in citric acid catabolism through *de novo* lipogenesis pathway. Downregulation of ACLY and FASN through AKT degradation together with CS direct inhibition resulted in an impressive response of gemcitabine in pancreatic cancer tumors. Moreover, a deregulated citrate metabolism correlated with drastic inhibition of LDHA expression in those tumors, possibly reducing microenvironment acidification and increasing gemcitabine availability for tumors.

The role of vitamin C as a metabolic modulator in PDAC highlights the importance of this molecule for the design of new strategies, in combination with gemcitabine and other chemotherapies, increasing the response of the tumor to treatments.

## Conflict of interest

The authors declare no conflict of interest.

## Author contributions

AC‐C, AG‐B devised and performed all the experiments. MJF‐A contributed and analyzed the immunohistochemical assays. NB‐H, LM‐V, LdP and AC designed the murine models. EN‐D, HP‐S, JAP, MC‐B and JAG performed modeling and kinetic assays. CM and JT performed glycolytic rate assays. AR‐V performed statistical analysis. OA and JG‐F conceived the project, supervised the experiments, and wrote the manuscript.

### Peer review

The peer review history for this article is available at https://www.webofscience.com/api/gateway/wos/peer‐review/10.1002/1878‐0261.13616.

## Supporting information


**Fig. S1.** FASN knockdown sensitizes PDAC cell lines to gemcitabine. A. TGCA analysis of *FASN* expression in tumor(red) vs healthy tissue (gray). B Western Blot showing FASN expression in pLKOemply and PlkoFASNshRNA transfected Mia‐Paca2 and CRL‐2558 cell lines (*n* = 3). B. FASN gene expression in pLKOemply and PlkoFASNshRNA transfected Mia‐Paca2 and CRL‐2558 cell lines (*n* = 3). C. Apoptosis analysis in pLKO and PlkoFASNshRNA in Mia‐Paca 2 and CRL‐2558 cell lines after 2.5 um and 5um GEM treatment for 48 h n = 3. Data are presented as mean ± s.e.m Statistical analyses were determined by one‐way ANOVA with Tukey's multiple comparison test (*P < 0.05; **P < 0,01; ***P < 0.001; ****P < 0.0001).

**Fig. S2.** Vitamin C augments mitochondrial pool. Mitochondrial marker VDAC1 expression after vitamin C (VC) treatment in Mia‐Paca 2 and CRL‐2558 cells. Data are presented as mean ± s.d. Statistical analyses were determined by two‐tailed unpaired t‐test (*P < 0.05; **P < 0,01; ***P < 0.001; ****P < 0.0001).

**Fig. S3.** NiCl_2_ stabilizes HIF1a. HIF1a protein expression upon 24 h NiCl_2_ exposure in Mia‐Paca 2 and CRL‐2558 cell lines. *n* = 3.

**Fig. S4.** Citrate metabolism correlates with glycolysis in pancreas. A. TGCA and GTEX data‐based Pearson correlation analysis of de citrate metabolism signature vs glycolysis signature. B. LDHA and PDK1 protein expression after 6 h vitamin C (VC) treatment in Mia‐Paca 2 and CRL‐2558 cell lines (*n* = 3). C*. LDHA* and *PDK1* mRNA expression upon 6 h ascorbate (VC) exposure in Mia‐Paca 2 and CRL‐2558 cell lines (*n* = 3). Data are represented as mean ± s.d. Statistical analysis was carried out using unpaired t‐test with Welch's correction.

## Data Availability

All reagents generated in this study are available from the lead contact, Óscar Aguilera Martinez (oscar.aguilera@quironsalud.es), without restriction.
