# Supplementary figures and images for: Vitamin‐C‐dependent downregulation of the citrate metabolism pathway potentiates pancreatic ductal adenocarcinoma growth arrest
Source: Mol Oncol. 2024 Feb 29;18(9):2212–33. doi: 10.1002/1878-0261.13616 (PMC11467799; doi:10.1002/1878-0261.13616)

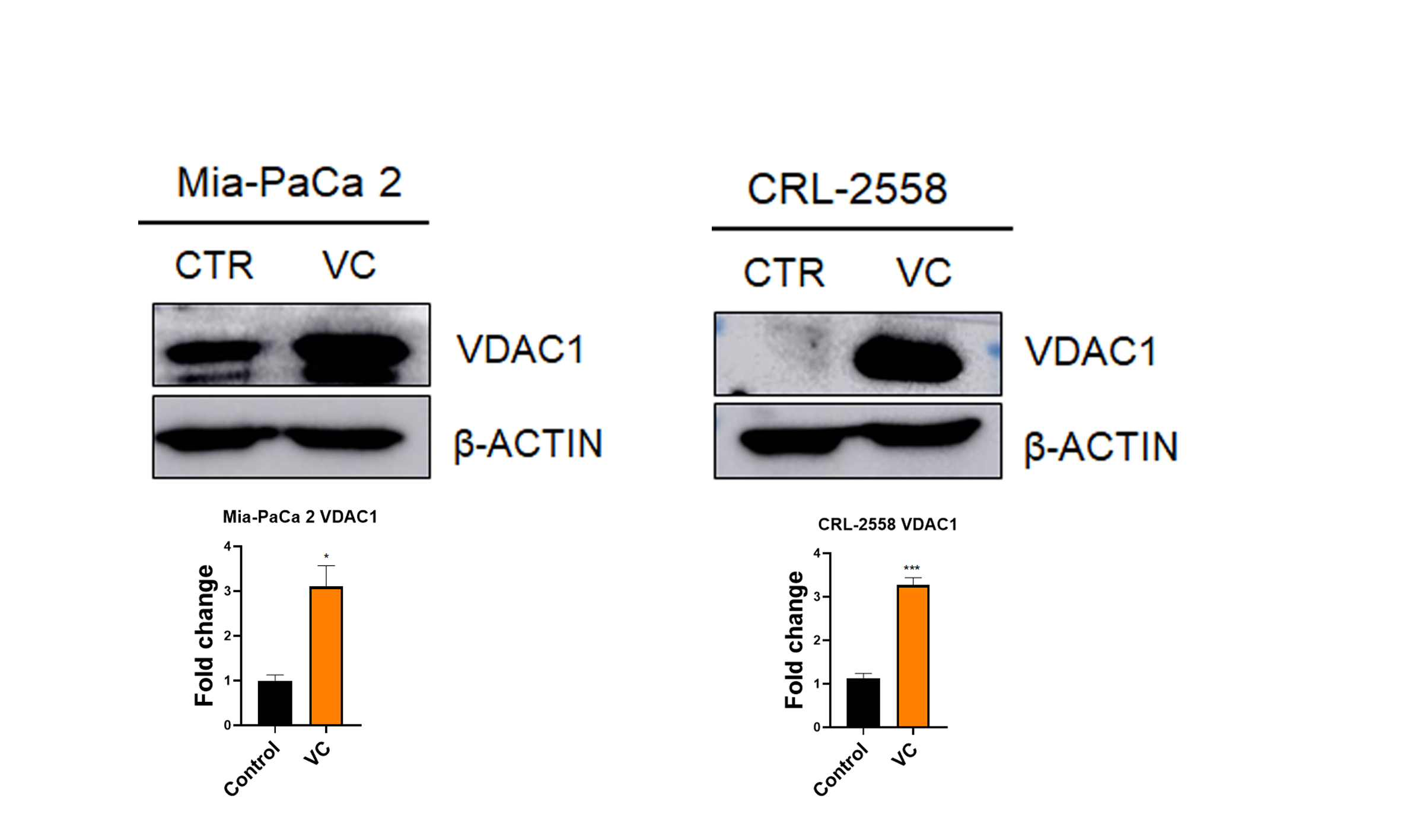

Supplement: Supplementary file 2 — Fig. S2. Vitamin C augments mitochondrial pool. Mitochondrial marker VDAC1 expression after vitamin C (VC) treatment in Mia‐Paca 2 and CRL‐2558 cells. Data are presented as mean ± s.d. Statistical analyses were determined by two‐tailed unpaired t‐test (*P < 0.05; **P < 0,01; ***P < 0.001; ****P < 0.0001). [file MOL2-18-2212-s001.tif]

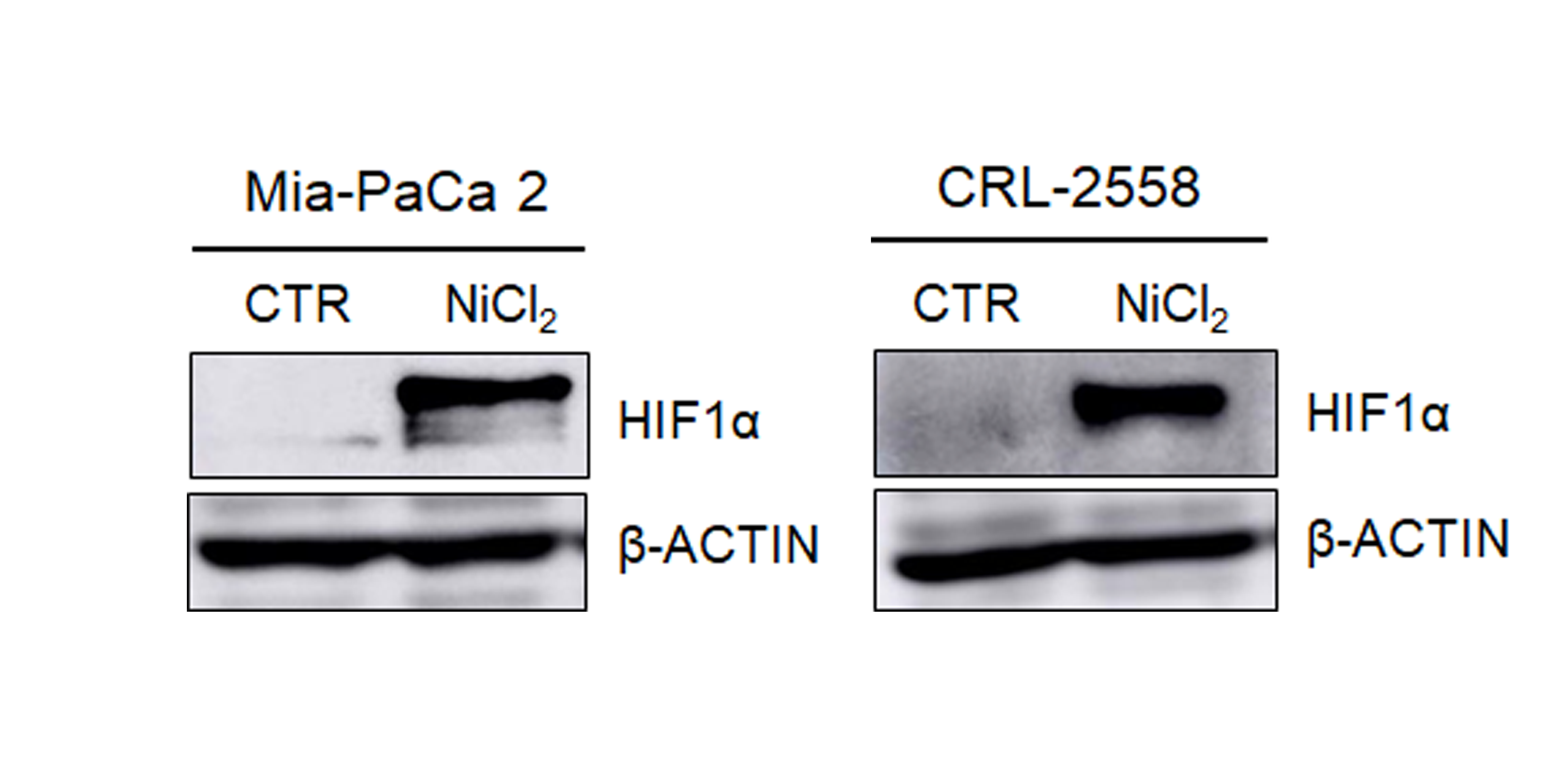

Supplement: Supplementary file 3 — Fig. S3. NiCl2 stabilizes HIF1a. HIF1a protein expression upon 24 h NiCl2 exposure in Mia‐Paca 2 and CRL‐2558 cell lines. n = 3. [file MOL2-18-2212-s003.tif]
